# A Systematic Literature Review of Adverse Events Associated with Systemic Treatments Used in Advanced Soft Tissue Sarcoma

**DOI:** 10.1155/2016/3597609

**Published:** 2016-07-19

**Authors:** Ann Colosia, Shahnaz Khan, Michelle D. Hackshaw, Alan Oglesby, James A. Kaye, Jeffrey M. Skolnik

**Affiliations:** ^1^Market Access and Outcomes Strategy, RTI Health Solutions, 200 Park Offices Drive, Research Triangle Park, Durham, NC 27709, USA; ^2^US Health Outcomes, Oncology, GlaxoSmithKline, 5 Crescent Drive, Philadelphia, PA 19112, USA; ^3^Epidemiology, RTI Health Solutions, 1440 Main Street, Suite 310, Waltham, MA 02451, USA; ^4^US Medical Affairs, Oncology, GlaxoSmithKline, 5 Crescent Drive, Philadelphia, PA 19112, USA

## Abstract

This systematic literature review describes adverse events (AEs) among patients with soft tissue sarcoma (STS) who received second-line or later anticancer therapies. Searches were conducted in PubMed, EMBASE, and Cochrane Central Register of Controlled Trials for studies of adults with advanced or metastatic STS who received systemic anticancer therapy before enrollment in a randomized-controlled trial of pazopanib, another targeted cancer agent, or cytotoxic chemotherapy. Of 204 publications identified, seven articles representing six unique studies met inclusion criteria. Additional safety results for pazopanib were identified on ClinicalTrials.gov. Hematologic toxicities were common with all therapies evaluated (pazopanib, trabectedin, dacarbazine ± gemcitabine, gemcitabine ± docetaxel, cyclophosphamide, and ifosfamide). Studies differed in AE type, timing of assessment, and outcomes reported, although patient populations and AE assessment timing were relatively similar for pazopanib and trabectedin. AEs that were more common with trabectedin than pazopanib were anemia, neutropenia, nausea/vomiting, and elevations in aspartate aminotransferase and alanine aminotransferase. An AE that was more common with pazopanib than trabectedin was anorexia. Only the pazopanib study reported AE frequencies versus placebo. A planned meta-analysis was not feasible, as there was no common comparator. More well-designed studies that include common comparators are needed for comparison of safety effects among treatments for STS.

## 1. Introduction

Soft tissue sarcomas (STS) are malignant tumors that begin in any of the mesodermal tissues of the extremities, trunk, retroperitoneum, or head and neck [1] and include more than 50 histologic subtypes [2]. In 2014, it was estimated that there would be 12,020 new cases of STS and 4740 deaths from STS in the United States (US) [3]. The overall estimated 5-year survival rate is 65.3% in the US, and the 5-year survival is 18.4% in patients with sarcomas with distant spread [3].

Treatment options for STS include surgery, radiotherapy, and systemic anticancer therapy (cytotoxic chemotherapy or targeted cancer agents). Surgery and radiotherapy are the standard initial treatment options for patients with primary resectable STS; however, up to 50% of patients experience recurrence [4]. For patients with advanced, unresectable, or metastatic STS, chemotherapy is the mainstay of treatment. Widely used cytotoxic chemotherapy regimens include dacarbazine, doxorubicin, epirubicin, and ifosfamide as single agents and anthracycline-based combinations (e.g., doxorubicin or epirubicin with ifosfamide, with or without dacarbazine). The National Comprehensive Cancer Network [5] and the European Society of Medical Oncologists [6] recommend an anthracycline (alone or in combination with other agents) as first-line treatment for metastatic STS in most cases, although first-line treatment recommendations may vary by histologic subtype and previous treatment. Other cytotoxic chemotherapy agents that have shown activity in clinical trials are gemcitabine, docetaxel, vinorelbine, pegylated liposomal doxorubicin, temozolomide [5], and trabectedin [6]. All of these agents can be associated with significant adverse events (AEs), including pancytopenia, febrile neutropenia, nausea, alopecia, and fatigue. Some long-term AEs may occur, including cardiomyopathy with doxorubicin and other anthracyclines [4].

Recently, a number of targeted cancer agents, including imatinib, sunitinib, and pazopanib, have demonstrated activity in particular STS histologic subtypes [5]. Imatinib is a tyrosine kinase inhibitor approved for treating gastrointestinal stromal tumors, but it failed to show activity in other histologic subtypes of STS [7]. Sunitinib, a multityrosine kinase inhibitor of vascular endothelial growth factor receptor- (VEGFR-) 2, platelet-derived growth factor receptor- (PDGFR-) *β*, and c-Kit, showed activity in patients with locally advanced or metastatic STS in a nonrandomized phase II trial [8]. Pazopanib is a multityrosine kinase inhibitor of VEGFR-1, VEGFR-2, VEGFR-3, PDGFR-*α*, PDGFR-*β*, and c-Kit. Pazopanib and trabectedin are the only therapies approved in the US for use as monotherapy for the treatment of patients with advanced STS who have received prior chemotherapy [9] (although trabectedin was not approved in the US at the time of the systematic literature review described in this article). Outside the US, trabectedin is approved for treatment in advanced STS after failure of anthracycline and ifosfamide or in patients with advanced STS for whom these agents are not suitable [10]. In the US, it is indicated for patients with advanced liposarcoma or leiomyosarcoma after an anthracycline-containing regimen.

In general, symptom palliation is the goal of treatment with cytotoxic chemotherapy and targeted cancer agents in patients with metastatic disease. There is a paucity of published data from randomized controlled trials (RCTs) evaluating these therapies compared with best supportive care, and no studies of targeted agents have demonstrated a survival advantage in metastatic STS to date [4]. Given the palliative nature of the therapy, lower toxicity regimens are desirable in this population.

There is no single data source that addresses the broad range of AEs associated with systemic anticancer therapy, that is, cytotoxic chemotherapy (classic chemotherapy agents such as doxorubicin and ifosfamide) or targeted cancer agents (drugs developed to target a specific protein in cancer cells such as pazopanib) in STS. To address this gap and to better understand the safety profile of these agents in this disease, we conducted a systematic literature review to review the tolerability and associated toxicities of pazopanib and other targeted cancer agents and cytotoxic chemotherapies used in the treatment of advanced or metastatic STS. A subsequent meta-analysis, if feasible, was planned to compare information on reported AEs associated with pazopanib and other therapies in the management and treatment of STS.

## 2. Materials and Methods

A systematic literature review was conducted on April 9, 2014, in accordance with the Preferred Reporting Items for Systematic Reviews and Meta-Analyses (PRISMA) guidelines [19]. PubMed, EMBASE, and the Cochrane Central Register of Controlled Trials (CENTRAL) were searched in order to qualitatively assess the frequencies of specific AEs occurring in adult patients with a diagnosis of advanced or metastatic STS other than gastrointestinal stromal tumors (since they are biologically distinct from other STS subtypes regarding their targeted treatment) who received prior systemic anticancer therapy from RCTs, as well as to assess the feasibility of conducting a meta-analysis of identified placebo-controlled trials. There were no date limits, but only publications in English were included. The study (ID: 201358) and protocol can be found at http://www.gsk-clinicalstudyregister.com/study/201358#ps. An example of the search strategies employed is presented in Additional file 1: Table S1 in Supplementary Material available online at http://dx.doi.org/10.1155/2016/3597609.

Bibliographies of included articles were reviewed for additional relevant studies not identified in the electronic database search. Included studies were RCTs in adults (aged ≥18 years) with a diagnosis of advanced/metastatic STS who had received at least one line of systemic anticancer therapy before enrollment in an RCT of pazopanib or another targeted cancer agent or cytotoxic chemotherapy. The original protocol for this systematic literature review included only studies that were blinded, even if only single-blinded. The goal of this inclusion criterion was to reduce bias on the part of patients and/or investigators in the reporting of AEs. After completing level 2 screening (see Section 2.2), we determined that only one study met all of the inclusion criteria (a study of pazopanib by van der Graaf et al. [11]). Because several other studies violated the inclusion criteria only by being open-label, the protocol was amended to include open-label studies to allow a qualitative review of the literature. Other inclusion criteria were that at least one arm of the studies had to evaluate a drug of interest and report an AE of interest.

A list of AEs of interest (Additional file 2: Table S2) was initially developed before conducting the literature review by reviewing product labels, and we further modified this list based on desktop research and clinical expertise. The primary endpoint included any frequency of any grade of the AEs, including separate reporting of grade 3 and/or 4 frequencies. If a meta-analysis had been feasible, the secondary endpoints were to be the same AEs. Studies in which all or most of the patients had gastrointestinal stromal tumor, childhood sarcomas, and other sarcomas or related tumors with unique treatment approaches were excluded.

### 2.1. Data Extraction

Data extracted from the eligible studies included trial characteristics, treatment information (dosing regimen), patient demographics, descriptions of the AEs captured (e.g., on-treatment only or a specified period beyond treatment, AEs for the intent-to-treat population or only for patients who actually received treatment, most common AE, and AEs occurring in ≥*X*% of patients), and data for the selected endpoints wherever available. Additionally, the number of patients experiencing any grade of an AE and the total number of patients in the safety population for that treatment arm were captured. Data were extracted by one reviewer, and the accuracy was checked by a second reviewer.

### 2.2. Quality Control and Assessment

Quality control procedures for inclusion and exclusion of articles included level 1 (titles/abstracts) and level 2 (full-text) screening for eligibility according to the inclusion and exclusion criteria, which were performed independently by two researchers. Articles for which there was any uncertainty about inclusion were discussed with a third researcher. Data were extracted from full-text versions of articles. Resources obtained via the Internet, such as results pages from ClinicalTrials.gov and the Food and Drug Administration Oncologic Drugs Advisory Committee Briefing Document, were saved as PDF files to maintain a record of information in case the electronic source was changed or removed. Quality control procedures for the data extraction included verification by a second researcher of all extracted data with original sources. The quality assessment of evidence from RCTs was based on guidance in the National Institute for Health and Care Excellence single technology appraisal* Specification for Manufacturer/Sponsor Submission of Evidence* [20] and adapted from the Centre for Reviews and Dissemination's guidance for undertaking reviews in health care [21].

### 2.3. Qualitative Data Synthesis

The qualitative assessment of RCTs identified in the systematic literature review did not involve statistical methods. Results are described qualitatively with more detailed results presented in supporting tables.

## 3. Results and Discussion

### 3.1. Search Results

**Figure 1:**
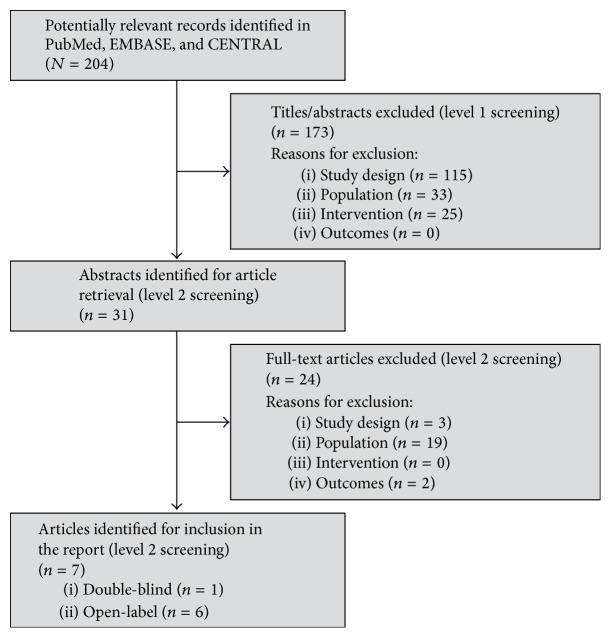
PRISMA flow diagram, Amendment 1. CENTRAL: Cochrane Central Register of Controlled Trials; PRISMA: Preferred Reporting Items for Systematic Reviews and Meta-Analyses.

Figure 1 shows the PRISMA flow diagram [22], which documents the number of articles excluded after title/abstract review and full-text review and the number of articles ultimately meeting the inclusion criteria after protocol amendment.

Searches of PubMed, EMBASE, and CENTRAL identified 204 studies after removal of duplicates. The original protocol called for inclusion of RCTs that were single-, double-, or triple-blinded. However, only the pazopanib study by van der Graaf et al. [11] met all of the inclusion criteria. With only one placebo-controlled study, a meta-analysis was not feasible.

The qualitative review was not restricted to placebo-controlled studies and RCTs with active comparators could be included. During level 2 review, five articles representing four open-label RCTs with active comparator arms were identified that met all inclusion criteria for the qualitative review except for blinding (Table 1; Additional file 3: Table S3) [12–14, 16, 17]. Pautier et al. [17] included two multicenter, open-label, phase II studies; patients were stratified by uterine and nonuterine sites of origin of leiomyosarcoma into two distinct phase II studies conducted in one trial. Based on the protocol amendment, these four studies could be included in the qualitative safety analysis.

The protocol amendment required revisiting the open-label studies excluded at level 1 review. Four open-label studies were identified [15, 23–25]. The full-text articles for these studies were reviewed to determine their eligibility based on the amended protocol, and only one was eligible for inclusion in the qualitative review (a study by Demetri and colleagues [15]).

### 3.2. Included Studies

A total of six studies from the 7 publications (Bramwell et al. 1986, 1987, and 1993 represented 1 study and Pautier et al. represented 2 studies) were included in this review: one double-blind placebo-controlled study [11] and five open-label RCTs [12–17]. All six studies were multicenter (Table 1; Additional file 3: Table S3): three studies were multinational [11, 12, 15] and three were conducted in only one country [16, 17]. There was one phase III study [11], and the remaining studies were all phase II [12, 15–17]. The treatment arm sizes ranged from 22 to 239. The studies with the largest patient populations were those evaluating pazopanib (*n* = 369) [11] and trabectedin (*n* = 270) [15].

The drugs assessed in the trials included pazopanib versus placebo [11]; cyclophosphamide versus ifosfamide (one study in three reports) [12–14]; two different dosing schedules of trabectedin [15]; dacarbazine ± gemcitabine [16]; and gemcitabine ± docetaxel (two studies in one report) [17].

Predictably, the main source of risk of bias was lack of blinding in all included studies except the pazopanib study, which was double-blinded (Additional files 4–9: Tables S4–S9) [11]. The appropriateness of the method of randomization was insufficiently described in four of the studies (in five reports) [12–14, 16, 17].

### 3.3. Cross Over

The studies differed in whether they allowed patients to cross over to the other study treatment after disease progression occurred on the initially assigned regimen. Patients were allowed to cross over to the other study drug in Bramwell et al. [12–14], which evaluated cyclophosphamide versus ifosfamide and in Demetri et al. [15], which evaluated different dosing schedules of trabectedin. In the study of pazopanib versus placebo by van der Graaf et al. [11], patients were not allowed to cross over when the disease progressed, but postprogression therapy included trabectedin, gemcitabine, taxanes, ifosfamide, dacarbazine, and antiangiogenic agents [26]. There was no discussion of crossover in the García-Del-Muro et al. study of dacarbazine ± gemcitabine [16] or the two studies of gemcitabine ± dacarbazine [17]. It was not explicitly stated that reported AEs were limited to the period prior to crossover, but we made this assumption when reporting the results. The main effect of crossover in clinical trials is to confound overall survival comparisons between randomized treatment groups, but overall survival was not the focus of this review.

### 3.4. Patient Populations

Most patients in all of the studies had metastatic rather than unresectable locally advanced disease (Additional file 10: Table S10). Although the Bramwell et al. study of cyclophosphamide versus ifosfamide allowed chemotherapy-naïve patients into the study, it presented a separate AE outcome (leukopenia) stratified by whether or not the patient had received previous chemotherapy [12–14].

The performance status of the populations in all of the studies did not differ widely. Although some studies allowed patients with a performance status of 2 to enroll [16, 17], most patients in all of the studies had a performance status of 0 or 1 (Additional file 10: Table S10). The median ages were similar in most of the studies (about 50–54 years) except for the nonuterine leiomyosarcoma group in the study by Pautier et al. [17], in which the median ages were 62 to 64 years for the gemcitabine ± docetaxel groups.

The predominant histology in all of the studies was leiomyosarcoma, representing 100% of the patients in the study of gemcitabine ± docetaxel by Pautier et al. [17] and 27% to 66% of the patients in the other studies. Patients with adipocyte/liposarcoma were excluded from the study of pazopanib [11], but they represented 3% to 34% of the patients in the studies of cyclophosphamide versus ifosfamide [13], dacarbazine ± gemcitabine [16], and trabectedin [15]. Except for the two studies including only patients with leiomyosarcoma (uterine and nonuterine) [17], 10% to 16% of patients had synovial sarcoma.

With the exception of the Pautier et al. study of uterine leiomyosarcoma [17], both sexes were well represented in the studies. Most or all of the patients in the reviewed studies had metastatic disease (versus unresectable locally advanced STS). The median ages were 40 to 64 years (Additional file 10: Table S10).

### 3.5. Previous Treatment

As a requirement for inclusion in this review, all studies presented data on patients who had received previous chemotherapy. Most or all of the patients were receiving the study drug as second-line therapy.

Only the Bramwell et al. [12] study evaluating cyclophosphamide versus ifosfamide enrolled patients with no previous chemotherapy, and separate AE data were presented for 42% of patients who had received previous chemotherapy. Of these 56 patients (42% of the total study population), 67% had received only one previous drug, which was typically an anthracycline, and 12.5% had received ≥3 drugs. However, the article did not report whether any of the ≥3 drugs were given in combination, which would result in fewer lines of therapy. It was also unclear whether the one drug (usually an anthracycline) was given as neoadjuvant, adjuvant, or first-line therapy for advanced disease. In the pazopanib study [11], 93% of patients had received previous systemic anticancer therapy for advanced disease and 56% had received ≥2 lines of treatment for advanced disease. Similarly, the trabectedin study required that patients had received previous anthracycline and ifosfamide (combined or sequentially) therapy, but it did not restrict this treatment to the advanced-disease setting, so some patients received the study drug as first-line therapy for advanced disease [15]. The median number of previous regimens for advanced disease was one, so at least one-half of the patients in this study received trabectedin as second- or later-line therapy, with some receiving it as up to seventh-line therapy. Also, approximately one-third of the patients had received agents not approved by a regulatory agency for advanced STS, including gemcitabine, docetaxel, and other investigational agents.

In the pazopanib study, 93% of patients had received previous systemic therapy for advanced STS, and 56% of patients received pazopanib as third- or later-line therapy [11]. Compared with the other studies, it appears that patients in the pazopanib study were more heavily pretreated, based on the number of previous systemic anticancer lines administered.

In the study of dacarbazine ± gemcitabine [16], all patients had received previous treatment with an anthracycline, ifosfamide, or both. The authors described the population as “heavily pretreated,” which seems to be variably defined with regard to cancer patients. For 77% of the patients in this study, disease progression occurred within 1 year of the prior therapy.

In the two studies of gemcitabine ± docetaxel [17], eligible patients had to receive only one prior doxorubicin-containing regimen. Most patients either received the doxorubicin-containing regimen as first-line therapy or, at disease progression within 1 year of adjuvant therapy, were considered to have received first-line doxorubicin. Few patients had an interval of >1 year after adjuvant therapy with anthracycline.

### 3.6. Methods of AE Reporting

The methods used for assessing safety in the trials varied (Additional file 11: Table S11). Three studies used the AE definitions from the National Cancer Institute (NCI) Common Terminology Criteria (CTC) [11, 16, 17] and one study used the* Medical Dictionary for Regulatory Activities* definitions with grading of severity by NCI CTC [15]. Another study used World Health Organization grade toxicity [12–14], which was the only study that was clear about the time frame of safety assessment (“after the first course and throughout treatment”).

The pazopanib study reported treatment-emergent AEs [11, 27], whereas the other studies did not specify whether all AEs or only treatment-emergent AEs were being reported. The study of dacarbazine ± gemcitabine presented only “clinically relevant toxicities” [16]. Leukopenia was the only AE reported separately for patients with previous chemotherapy experience in the study of cyclophosphamide versus ifosfamide in Bramwell et al. [12–14].

Only the Pautier et al. study of gemcitabine ± docetaxel did not report safety outcomes as the number and percentage of patients experiencing the AE [17]. Instead, this study reported the “percentage of cycles for which patients experienced toxicity.” The trabectedin study presented both types of outcomes [15].

This report uses “frequency” to mean the percentage of patients experiencing a particular AE as the worst grade occurrence or the percentage of patients experiencing AEs per cycle. “Rate” is not appropriate for comparisons among studies as the time frame for AE assessment presumably varied because patients were treated for differing amounts of time, as deduced from the median number of cycles administered (Additional file 11: Table S11).

### 3.7. Adverse Events

As previously mentioned, leukopenia was the only AE reported for patients with previous chemotherapy in the study of cyclophosphamide versus ifosfamide [12–14]. Only two other studies reported leukopenia [11, 16, 18], so this AE is summarized separately from the remaining AEs. In the pazopanib trial, grade 3 leukopenia occurred in three patients (1%) receiving pazopanib and no patients (0%) receiving placebo [11]. Grade 3 and 4 leukopenia occurred in higher percentages of patients treated with cyclophosphamide than with ifosfamide [12–14]. The Bramwell et al. study also included chemotherapy-naïve patients, and leukopenia was worse in patients with previous chemotherapy. The occurrence of serious infections was similar (~7%) in both treatment groups for all patients (both with and without previous chemotherapy). Leukopenia occurred in more patients receiving gemcitabine + dacarbazine than with dacarbazine alone, but grade 4 leukopenia was observed only in patients receiving dacarbazine alone [16].

An AE comparison of the studies reporting the percentage of patients experiencing AEs of interest [11, 15, 16, 18] is shown in Tables 2 [Table tab3] [Table tab4]–5. In the pazopanib study, AEs that occurred at a higher frequency with pazopanib than placebo were neutropenia, thrombocytopenia, aspartate aminotransferase (AST) elevation, alanine aminotransferase (ALT) elevation, bilirubin elevation, anorexia, weight loss, diarrhea, nausea/vomiting, dysgeusia, mucositis, fatigue, and hypertension [11, 18]. Venous thromboembolic events occurred in a small proportion of patients in both the pazopanib and placebo groups, with a higher frequency in the pazopanib group.

Between the two trabectedin schedules, most on-treatment laboratory abnormalities occurred at a higher frequency with the every 3-week 24-hour infusion schedule than with the weekly 3-hour infusion schedule [15]. These AEs included the nonhematologic AEs elevations in AST, ALT, and bilirubin as well as the hematologic AEs neutropenia and thrombocytopenia. Drug-related nonlaboratory AEs were generally similar between the two treatment groups, except that nausea/vomiting was more common with the every 3-week 24-hour infusion schedule, and dyspnea occurred more often with the less-efficacious weekly 3-hour schedule (28%) and occurred in 17% of patients receiving the recommended every 3-week 24-hour schedule.

In the García-Del-Muro et al. study assessing gemcitabine ± dacarbazine, only the hematologic AE thrombocytopenia occurred more frequently among the AEs reported as clinically relevant in the group receiving dacarbazine alone [16]. All other reported AEs were more common in the dacarbazine + gemcitabine arm, including the hematologic AEs anemia, febrile neutropenia, neutropenia, the nonhematologic AEs diarrhea, and nausea/vomiting.

When AE frequencies were reported by the percentage of cycles during which the AE occurred, the trabectedin every 3-week 24-hour infusion schedule continued to have higher frequencies than the weekly 3-hour infusion schedule [15]. However, bilirubin elevation was equal between the two groups, and constipation occurred at a higher frequency with the every 3-week 24-hour infusion schedule.

Alopecia, asthenia, and fluid retention all occurred in a higher percentage of cycles in patients receiving gemcitabine + docetaxel than in patients receiving gemcitabine monotherapy [17]. Except for few occurrences of grade 3/4 asthenia, these three AEs were grade 1/2 in severity. Fever/infections occurred in a similar percentage of cycles between the two treatment groups, and most of these occurrences were grade 1/2 in severity.

### 3.8. Liver-Related AEs

More patients in the pazopanib group experienced elevations in AST, ALT, and bilirubin than in the placebo group (Table 2) [11, 18]. The percentage of patients experiencing grade 3 and 4 AST, ALT, and bilirubin elevations was ≤10%, with clinical assessments of safety, including laboratory assessments, done at baseline, weeks 4, 8, and 12, and at 8-week intervals thereafter, and dose modifications possible for AEs.

Nearly all patients receiving trabectedin in the every 3-week 24-hour infusion schedule arm experienced AST or ALT elevations, and ≥32% had grade 3 elevations [15]. Grade 3 and 4 elevations in AST and ALT were noncumulative and transient with a median duration of elevation of 7 to 8 days. Grade 3 increases in AST or ALT were much higher in the 3-week 24-hour infusion schedule arm than in the weekly 3-hour infusion schedule (Table 2). Only one patient in each trabectedin arm had grade 3 bilirubin elevation, and no patients experienced grade 4 bilirubin elevation.

Liver enzyme and bilirubin elevations were not reported in the study of dacarbazine ± gemcitabine [16].

### 3.9. Gastrointestinal and Eating-Related AEs

Anorexia, weight loss, diarrhea, and nausea/vomiting were all common with pazopanib, occurring in 40%, 48%, 58%, and 54% of patients, respectively (Table 3) [11]. Anorexia was also common in patients receiving trabectedin administered via the every 3-week 24-hour infusion schedule (22%), as were diarrhea (24%) and nausea/vomiting (75%) [15]. In both the pazopanib and trabectedin trials, grade 3/4 events occurred at relatively low frequencies (≤6%). There were no occurrences of grade 3/4 weight loss with pazopanib [11]. Adding gemcitabine to dacarbazine increased the frequency of all-grade diarrhea and nausea/vomiting, but there were no grade 3/4 occurrences of diarrhea and similarly low frequencies (2%) of nausea/vomiting in the two treatment groups [16].

### 3.10. Mouth or Taste

Only the pazopanib study reported on dysgeusia (taste disorder) and mucositis (Table 3). Both were higher in the pazopanib group than in the placebo group. There were no grade 3/4 occurrences of dysgeusia and only three occurrences of grade 3 mucositis in the pazopanib group [11].

### 3.11. Other AEs

Alopecia was reported in the study of dacarbazine ± gemcitabine; this AE occurred at low frequencies (7% and 2%, resp.), and all occurrences were grade 2 in severity (Table 4) [17]. The frequency of asthenia of any grade increased with the addition of gemcitabine, but grade 3 asthenia frequencies were similar between the two groups [16]. Cough was reported as a drug-related AE in 17% and 18% of patients receiving trabectedin at the weekly 3-hour and the every 3-week 24-hour schedules, respectively [15]. Dyspnea occurred more often with the weekly 3-hour schedule (28%) of trabectedin than with the recommended every 3-week 24-hour schedule (17%).

Embolism of any grade occurred at a low frequency (≤5%) but occurred more often with pazopanib than with placebo [11]. Fatigue was common with either schedule of trabectedin (68%–75%) and was mostly grade 1/2 [15]. Fatigue was also common with pazopanib (65%), but 49% of patients receiving placebo also reported fatigue [11]. Hypertension, primarily grade 1/2, occurred in 41% of patients receiving pazopanib and 7% of patients receiving placebo.

### 3.12. Hematologic AEs

For hematologic AEs, patients receiving pazopanib had only a slightly higher frequency of treatment-emergent anemia than patients receiving placebo (Table 5) [18]. The frequencies of all-grade anemia in the pazopanib study were 27% and 23% with pazopanib and placebo, respectively. The frequency of all-grade neutropenia was 33% in patients receiving pazopanib and 7% in patients receiving placebo. The frequency of febrile neutropenia by grade was not reported in the pazopanib study. Thrombocytopenia occurred more frequently in patients receiving pazopanib versus placebo.

For trabectedin, anemia was common with both schedules, but approximately one-half of patients entering the study had preexisting anemia [15]. Neutropenia was the most common grade 3/4 hematologic toxicity and was more common with the every 3-week 24-hour infusion (47%) than with the weekly 3-hour infusion schedule (13%). Grade 4 neutropenia with trabectedin was of short duration, and febrile neutropenia occurred in <1% of patients treated with trabectedin. Thrombocytopenia occurred more frequently in patients receiving trabectedin by the every 3-week 24-hour infusion schedule than by the weekly 3-hour infusion schedule.

Febrile neutropenia occurred in <10% of patients in the dacarbazine ± gemcitabine study [16]. Thrombocytopenia occurred more frequently in patients receiving dacarbazine alone versus dacarbazine + gemcitabine. This finding was true for all grades, grade 3, and grade 4 of thrombocytopenia.

## 4. Discussion

The goal of this systematic review was to compare a broad array of common AEs that affect patients' health and quality of life based on a list of prespecified AEs, developed by initially reviewing product labels and then modified based on desktop research and clinical expertise. An ideal outcome of this systematic literature review would have been a quantitative comparative assessment of pazopanib with other agents as second or later lines of treatment in previously treated patients with advanced or metastatic STS. However, such a comparison was not possible because there were no common comparators in the studies identified. The only randomized placebo-controlled study was that of pazopanib [11].

Only six RCTs in patients with STS who received previous systemic anticancer therapy met the inclusion criteria for this systematic review (adult patients, randomized design, and previous systemic anticancer therapies for STS), underscoring the paucity of medical evidence available in treating these diseases in the advanced or metastatic setting after first-line therapy. The median overall survival of patients in most arms of these studies was ≤1.5 years [11, 14, 15], with the exception of patients with uterine sarcoma who had a median survival of 20 and 23 months in the gemcitabine and gemcitabine + docetaxel groups, respectively [17]. The short life expectancy of patients in this setting underscores the need for tolerable later-line systemic anticancer therapies.

In addition to the lack of common comparators, comparisons would only be meaningful if the studies had similar patient populations and AE-reporting methods. Although gemcitabine was used in three studies, it was combined with a unique comparator in a study with dacarbazine [16]. The other two studies differed by anatomic location of leiomyosarcoma and were reported together, so the AEs of gemcitabine ± docetaxel were compared within the report [17]. Comparing gemcitabine + dacarbazine with gemcitabine + docetaxel was not possible, because the type of AE reported was not consistent; García-Del-Muro and colleagues reported the percentage of patients with AEs [16], whereas Pautier and colleagues reported the percentage of cycles with AEs [17].

With these limitations in mind, hematologic toxicities were common in the trials of pazopanib, trabectedin, dacarbazine ± gemcitabine, and gemcitabine ± docetaxel. High frequencies of grade 3/4 myelosuppression were observed with trabectedin [15] and dacarbazine + gemcitabine [16]. The toxicity profile of trabectedin and dacarbazine reported here is consistent with those from a phase III trial recently reported by Demetri et al. [28]. For trabectedin versus dacarbazine, rates of neutropenia were 49% versus 29% and rates of thrombocytopenia were 30% versus 36%. In the trabectedin arm, 45% experienced increased ALT levels versus 6% in the dacarbazine arm. In a separate phase III study in the first-line setting, trabectedin plus doxorubicin led to higher rates of grade 3 or 4 thrombocytopenia and liver toxicity, with no improvement in survival compared with doxorubicin alone [29]. Cyclophosphamide produced exceptionally high frequencies of grade 3/4 leukopenia, the only AE reported with higher rates in patients receiving cyclophosphamide, especially those who had prior chemotherapy, in the study by Bramwell et al. [12–14]. In our indirect comparison, grade 4 neutropenia and grade 3 thrombocytopenia were more frequent with trabectedin than pazopanib [11, 15]. Anorexia was more common in the pazopanib trial [11], but both pazopanib and trabectedin were associated with fatigue and elevations in AST and ALT in the two respective trials [11, 15].

Most studies in this review reported AE frequencies as percentages of patients [11–16]. It is unclear whether there is an advantage to reporting the percentage of cycles with a particular AE, and it suggests that the number of patients affected is obscured by the calculation. When AEs are reported by the percentage of patients at risk, the patient is counted once by the worst grade of a particular AE. An additional difficulty is that neither method tells anything about the timing of the AEs (e.g., whether an AE occurred early in treatment and then waned or worsened).

Reporting of the types of AEs was also variable in other ways, impairing comparability. The hematologic AEs in the six studies in this review appear to all to be worst grade while on treatment and therefore would be comparable if the time period of AE assessment and the patient population among studies were similar. Comparison of nonhematologic AEs is made difficult by the variation in the types reported: treatment-emergent (pazopanib) [11], drug-related (trabectedin) [15], and “clinically relevant” (gemcitabine ± dacarbazine) [16]. Besides the type of AE reported, the six studies in this review also differed or appeared to differ in the timing of AE assessments. The ifosfamide versus cyclophosphamide study noted that toxicity was assessed after the first course and throughout treatment but, like the remaining studies, reported only the median number of cycles. Pazopanib was taken orally every day in the clinical trials [11], whereas trabectedin, gemcitabine, dacarbazine, docetaxel, ifosfamide, and cyclophosphamide were all administered intravenously and periodically [12–17]. It is possible that the authors considered reporting the median number of cycles and range as a surrogate for duration of AE assessment. If that is the case, the every 3-week 24-hour infusion schedule in the trabectedin study and both the gemcitabine and gemcitabine + docetaxel arms may have had assessment periods similar to that for pazopanib, with median 3- to 4-week cycles of 5, 5, and 4, respectively [11, 15, 17]. Trabectedin every week, gemcitabine ± dacarbazine, cyclophosphamide, and ifosfamide may have had shorter AE assessment periods [12–16].

Of note, elevations in transaminases and bilirubin were not reported in the studies other than those for pazopanib and trabectedin. However, US labeling for dacarbazine [30] and gemcitabine [31] carry warnings of hepatic toxicity and directives to monitor hepatic function. The dacarbazine label notes that the number of incidences resulting in death is low, and death is mostly observed when dacarbazine is used in combination with other chemotherapy agents. Gemcitabine therapy in other cancers shows high frequencies of all-grade increases in AST and ALT when given as monotherapy, including grade 3/4 frequencies of approximately 10%. Increases in bilirubin were much less common (all grades, 13%) [31].

Cancer type may also have an effect on the frequency of AEs, as may prior therapy. For example, fatigue, nausea, anorexia, weight loss, and dysgeusia appear to occur more frequently in patients receiving pazopanib for STS than for other labeled indications for pazopanib, specifically renal cell carcinoma [11, 32]. In addition, the AEs in patients with nonadipocyte/nonliposarcoma STS receiving pazopanib were primarily grade 1/2 in severity and are AEs commonly managed in clinical practice [11]. Whether prior therapy, disease type, or other factors influence the frequency of reported AEs in clinical trials is unknown.

An earlier systematic literature review on the efficacy and safety of second- or later-line therapy in advanced or metastatic STS summarized grade 3/4, but not all-grade, safety outcomes from RCTs and single-arm studies of patients with advanced STS previously treated with an anthracycline and/or ifosfamide [33]. Of the randomized studies from the Sharma et al. review, five were also identified in this literature review [11, 15–17]. Two of the seven randomized studies in the review by Sharma and colleagues did not meet our inclusion criteria (Pacey et al., 2011: AEs not separated by treatment group; van Oosterom et al., 2002: not powered for comparison of the treatment groups) [34, 35]. We included an additional randomized study [12–14] not included in the Sharma et al. review, possibly because data were not presented separately by histologic subtype. As also found in the review summarized in this report, Sharma and colleagues note the lack of consistency with which AEs were reported among the RCTs [33]. The review by Sharma et al. notes the difference in grade 3/4 AE frequencies in patients treated with pazopanib compared to cytotoxic chemotherapies, the latter being associated with a higher occurrence of grade 3/4 hematologic toxicities [33].

Since our search of the literature, eribulin, a microtubule-dynamics inhibitor, was approved in 2016 for use as a single agent to treat liposarcoma [36]. Although not included in our study, it is worth mentioning that data from phase III trials evaluating eribulin and another agent, ombrabulin, in the relapsed setting have been published [37, 38]. Compared with dacarbazine, eribulin led to lower rates of thrombocytopenia (6% versus 28%) but higher rates of neutropenia (45% versus 24%) and leukopenia (16% versus 10%), and it was associated with peripheral sensory neuropathy (21% versus 4%) [38]. Ombrabulin, a tubulin-depolymerizing tumor vascular-disrupting drug, plus cisplatin was compared with cisplatin plus placebo [37]. Although there was a significant improvement in progression-free survival with ombrabulin, more patients in the ombrabulin group had grade 3 and 4 neutropenia (31% versus 9%) and thrombocytopenia (12% versus 9%).

### 4.1. Limitations

The major limitation of this review is the small number of randomized trials in previously treated patients with advanced or metastatic STS. Only the study of pazopanib was a phase III study; the remaining studies were phase II trials, a distinction that is perhaps less important in cancer research than the size of a trial. Phase III pazopanib and phase II trabectedin studies were both large (*n* = 369 and *n* = 270, resp.) [11, 15]. The population of the phase II study of cyclophosphamide versus ifosfamide was also considerable (*n* = 171) [12–14], but only a subset of patients had received previous chemotherapy and was eligible for inclusion in this review. With small treatment arm sizes (range, 22 to 57) in most of the studies in this review, common AEs would be detected, but the true risk of infrequent AEs would not be estimated very accurately, and rare AEs (e.g., those occurring in <1% of patients) might not be observed at all.

The quality of the studies was generally acceptable (Additional files 4–9: Tables S4–S9). The open-label design of all but the pazopanib study created a potential bias in the reporting of AEs. However, for practical reasons, many studies in late-stage cancer are open-label, and the risk of bias is tolerated to allow a qualitative review of salvage therapy for advanced or metastatic STS. Additionally, not all patients in the Pautier et al. study received anthracycline-based chemotherapy for metastatic disease [17]. In the nonuterine leiomyosarcoma group, five patients in the gemcitabine-only arm and four patients in the gemcitabine + docetaxel arm had not received first-line anthracycline-based chemotherapy for metastatic disease. In the uterine group, one patient in the gemcitabine arm and seven patients in the gemcitabine + docetaxel arm had not received anthracycline-based chemotherapy for metastatic disease [17].

Other limitations were noted above: the limited amount of safety information reported, including types of AEs; time period of assessment; and the variation in presentation of AEs (percentage of patients versus percentage of cycles). Finally, and very importantly, the limitations inherent in comparing across studies, across years that were a priori not prospectively designed to compare reported AEs by methodology, imply that any comparison of these studies should be made cautiously. However, given the limitations above for an exercise such as this, it is likely that, outside of an RCT, this is the best approach to assessing the frequency and severity of AEs in patients with STS who progress following first-line therapy.

## 5. Conclusions

Differences in the extent of previous systemic anticancer therapy and the types and timing of AEs reported precluded qualitative comparison of pazopanib with most of the interventions in the six studies in this review. Only the study of trabectedin was reasonably similar in these aspects [15]. A meta-analysis of safety endpoints was not feasible as there was no common comparator allowing for indirect analysis. The AEs that were more common with trabectedin on an every 3-week 24-hour infusion schedule than with pazopanib were all-grade and grade 3/4 anemia and neutropenia, all-grade nausea/vomiting, and all-grade and grade 3 elevations in AST and ALT. The AEs that were more common with pazopanib than with trabectedin were all-grade and grade 3/4 anorexia. More well-designed studies that include one or more comparators in common with the trials identified here are needed to provide additional medical evidence for the best treatment for STS in the advanced and/or metastatic setting.

## Supplementary Material

The supplemental appendix includes specific information on the literature search strategy, adverse events of interest for the comparisons, details of the identified studies included in the review, and a quality assessments of each of the articles. Also included are details of the patient characteristics of each study and the criteria used in each study to assess adverse events.

## Figures and Tables

**Table 1:** Characteristics of included studies after protocol amendment.

Reference	Phase	Randomized, *n*	Treatment	Patient group for safety	*n*	Median number of cycles (range)
van der Graaf et al., 2012 [11]	III	369	Pazopanib	Treated	239	Median treatment duration, 16.4 weeks (0–79 weeks)
			Placebo		123	Median treatment duration, 8.1 weeks (1–52 weeks)

Bramwell et al., 1986, 1987, 1993 [12–14]	II	171	Cyclophosphamide	Treated	29	2.5 (1–13)
			Ifosfamide		28	3 (1–15)

Demetri et al., 2009 [15]	II	270	Trabectedin 24 h IV every 3 weeks	Treated	130	5 (1–37)
			Trabectedin 3 h IV weekly for 3 of 4 weeks		130	2 (1–21)

García-Del-Muro et al., 2011 [16]	II	113	Dacarbazine	Treated and assessable	52	2 (1–10)
			Gemcitabine + dacarbazine		57	6 (2–12)

Pautier et al., 2012 [17]^*∗*^ Study 1: leiomyosarcoma	II	46	Gemcitabine	Treated	22	5
			Gemcitabine + docetaxel		24	

Study 2: nonuterine leiomyosarcoma	II	44	Gemcitabine	Treated	22	4
			Gemcitabine + docetaxel		22	

^*∗*^The article by Pautier et al. [17] presents the results of two independent phase II studies: one study in patients with uterine leiomyosarcoma and one in patients with nonuterine leiomyosarcoma. IV, intravenous.

**Table 2:** Patients experiencing selected liver-related AEs.

*Study*	van der Graaf et al., 2012 [11, 18]		Demetri et al., 2009 [15]		García-Del-Muro et al., 2011 [16]	

*Patient group*	Treated		ITT (independent review)		Treated and analyzed	

*Treatment group*	Placebo (*n* = 123)	Pazopanib (*n* = 239)	Trabectedin q3wk 24 h IV (*n* = 130)	Trabectedin weekly 3 h IV (*n* = 130)	Dacarbazine (*n* = 52)	Gemcitabine + dacarbazine (*n* = 57)

*Liver-related AEs, n (%)*						
AST elevation						
All grades	27 (22)^*∗*^	122 (51)^*∗*^	122 (94)	85 (65)	—	—
Grade 3	2 (2)^*∗*^	13 (5)^*∗*^	41 (32)	4 (3)	—	—
Grade 4	0 (0)^*∗*^	6 (3)^*∗*^	0 (0)	0 (0)	—	—
ALT elevation						
All grades	22 (18)^*∗*^	110 (46)^*∗*^	126 (97)	100 (77)	—	—
Grade 3	3 (2)^*∗*^	18 (8)^*∗*^	59 (45)	12 (9)	—	—
Grade 4	1 (1)^*∗*^	5 (2)^*∗*^	3 (2)	0 (0)	—	—
Bilirubin elevation						
All grades	9 (7)^*∗*,†^	68 (28)^*∗*,†^	28 (22)	15 (12)	—	—
Grade 3	2 (2)^*∗*,†^	3 (1)^*∗*,†^	1 (<1)	1 (<1)	—	—
Grade 4	0 (0)^*∗*,†^	0 (0)^*∗*,†^	0 (0)	0 (0)	—	—

^*∗*^These data were reported on ClinicalTrials.gov: NCT00753688.

^†^The number of patients with grade 3/4 bilirubin elevation differed slightly for the pazopanib group reporting in the article by van der Graaf et al. [11] and on ClinicalTrials.gov [18]. Because ClinicalTrials.gov had more information (all grades), this table shows the data from ClinicalTrials.gov.

AE: adverse event; ALT: alanine aminotransferase; AST: aspartate aminotransferase; ITT: intention to treat; IV: intravenous; q3wk: every 3 weeks.

**Table 3 tab3:** Patients experiencing selected gastrointestinal and/or eating-related AEs.

*Study*	van der Graaf et al., 2012 [11, 18]		Demetri et al., 2009 [15]		García-Del-Muro et al., 2011 [16]	

*Patient group*	Treated		ITT (independent review)		Treated and analyzed	

*Treatment group*	Placebo (*n* = 123)	Pazopanib (*n* = 239)	Trabectedin q3wk 24 h IV (*n* = 130)	Trabectedin weekly 3 h IV (*n* = 130)	Dacarbazine (*n* = 52)	Gemcitabine + dacarbazine (*n* = 57)

*Gastrointestinal and/or eating-related AEs, n (%)*						
Anorexia/decreased appetite						
All grades	24 (20)	95 (40)	29 (22)	21 (16)	—	—
Grade 3	0 (0)	14 (6)	—	—	—	—
Grade 4	0 (0)	0 (0)	—	—	—	—
Grade 3/4	0 (0)	14 (6)	1 (<1)	0 (0)	—	—
Constipation						
Grade 1/2	—	—	45 (35)	42 (32)	—	—
Grade 3/4	—	—	0 (0)	2 (2)	—	—
Decreased weight or weight loss						
All grades	25 (20)	115 (48)	—	—	—	—
Grade 3	0 (0)	0 (0)	—	—	—	—
Grade 4	0 (0)	0 (0)	—	—	—	—
Diarrhea						
All grades	20 (16)	138 (58)	31 (24)	28 (22)	3 (6)	10 (18)
Grade 3/4	1 (1)	11 (5)	1 (<1)	0 (0)	0 (0)	0 (0)
Nausea and/or vomiting						
All grades	34 (28)	129 (54)	97 (75)	67 (52)	8 (15)	23 (40)
Grade 3	2 (2)	8 (3)	—	—	1 (2)	1 (2)
Grade 4	0 (0)	0 (0)	—	—	—	—
Grade 3/4	2 (2)	8 (3)	7 (5)	3 (2)	—	—
*Mouth or taste*						
Dysgeusia						
All grades	5 (4)	64 (27)	—	—	—	—
Grade 3	0 (0)	0 (0)	—	—	—	—
Grade 4	0 (0)	0 (0)	—	—	—	—
Mucositis						
All grades	4 (3)	29 (12)	—	—	—	—
Grade 3	0 (0)	3 (1)	—	—	—	—
Grade 4	0 (0)	0 (0)	—	—	—	—

AE: adverse event; ITT: intention to treat; IV: intravenous; q3wk: every 3 weeks.

**Table 4 tab4:** Patients experiencing selected other AEs.

*Study*	van der Graaf et al., 2012 [11, 18]		Demetri et al., 2009 [15]		García-Del-Muro et al., 2011 [16]	

*Patient group*	Treated		ITT (independent review)		Treated and analyzed	

*Treatment group*	Placebo (*n* = 123)	Pazopanib (*n* = 239)	Trabectedin q3wk 24 h IV (*n* = 130)	Trabectedin weekly 3 h IV (*n* = 130)	Dacarbazine (*n* = 52)	Gemcitabine + dacarbazine (*n* = 57)

*Other AEs, n (%)*						
Alopecia						
Grade 1/2	—	—	—	—	1 (2)^*∗*^	4 (7)^*∗*^
Asthenia						
All grades	—	—	—	—	26 (50)	43 (76)
Grade 3	—	—	—	—	5 (10)	4 (7)
Grade 4	—	—	—	—	—	—
Cough						
All grades	—	—	23 (18)	22 (17)	—	—
Grade 3/4	—	—	0 (0)	1 (<1)	—	—
Dyspnea						
All grades	—	—	22 (17)	36 (28)	—	—
Grade 3/4	—	—	5 (4)	8 (6)	—	—
Embolism (including pulmonary and cerebrovascular)						
*Venous thromboembolic events*						
All grades	3 (2)	13 (5)	—	—	—	—
Fatigue						
All grades	60 (49)	155 (65)	97 (75)	89 (68)	—	—
Grade 3	6 (5)	30 (13)	—	—	—	—
Grade 4	1 (1)	1 (<1)	—	—	—	—
Grade 3/4	7 (6)	31 (13)	10 (8)	9 (7)	—	—
Headache						
All grades	—	—	37 (28)	34 (26)	—	—
Grade 3/4	—	—	1 (<1)	1 (<1)	—	—
Hypertension						
All grades	8 (7)	99 (41)	—	—	—	—
Grade 3	4 (3)	16 (7)	—	—	—	—
Grade 4	0 (0)	0 (0)	—	—	—	—

^*∗*^All grade 2.

AE: adverse event; ITT: intention to treat; IV: intravenous; q3wk: every 3 weeks.

**Table 5:** Patients experiencing selected hematologic AEs.

*Study*	van der Graaf et al., 2012 [11, 18]		Demetri et al., 2009 [15]		García-Del-Muro et al., 2011 [16]	

*Patient group*	Treated		ITT (independent review)		Treated and analyzed	

*Treatment group*	Placebo (*n* = 123)	Pazopanib (*n* = 239)	Trabectedin q3wk 24 h IV (*n* = 130)	Trabectedin weekly 3 h IV (*n* = 130)	Dacarbazine (*n* = 52)	Gemcitabine + dacarbazine (*n* = 57)

*Hematologic AEs, n (%)*						
Anemia						
All grades	28 (23)^*∗*^	65 (27)^*∗*^	126 (97)	117 (90)	34 (65)	47 (82)
Grade 3	1 (1)^*∗*^	11 (5)^*∗*^	5 (4)	9 (7)	4 (8)	2 (4)
Grade 4	1 (1)^*∗*^	4 (2)^*∗*^	5 (4)	3 (2)	2 (4)	0 (0)
Febrile neutropenia						
All grades	—	—	1 (<1)	1 (<1)	3 (6)	5 (9)
Grade 3	—	—	—	—	2 (4)	4 (7)
Grade 4	—	—	—	—	1 (2)	1 (2)
Neutropenia						
All grades	8 (7)^*∗*^	79 (33)^*∗*^	96 (74)	64 (49)	28 (53)	43 (76)
Grade 3	0 (0)^*∗*^	10 (4)^*∗*^	34 (26)	15 (12)	7 (13)	18 (32)
Grade 4	0 (0)^*∗*^	0 (0)^*∗*^	27 (21)	2 (2)	10 (19)	9 (16)
Grade 3/4	0 (0)^*∗*^	10 (4)^*∗*^	61 (47)	17 (13)	17 (32)	27 (48)
Thrombocytopenia						
All grades	7 (6)^*∗*^	86 (36)^*∗*^	70 (54)	36 (28)	31 (60)	23 (40)
Grade 3	0 (0)^*∗*^	7 (3)^*∗*^	12 (9)	6 (5)	8 (15)	1 (2)
Grade 4	0 (0)^*∗*^	2 (1)^*∗*^	3 (2)	1 (<1)	6 (12)	2 (4)

^*∗*^These data were reported on ClinicalTrials.gov: NCT00753688.

AE: adverse event; ITT: intention to treat; IV: intravenous; q3wk: every 3 weeks.

## Abbreviations

AE: Adverse event
ALT: Alanine aminotransferase
AST: Aspartate aminotransferase
CENTRAL: Cochrane Central Register of Controlled Trials
CTC: Common Terminology Criteria
CTCAE: Common Terminology Criteria for Adverse Events
ECOG: Eastern Cooperative Oncology Group
EORTC: European Organisation for Research and Treatment of Cancer
G-CSF: Granulocyte-specific colony-stimulating factor
ITT: Intent to treat
IV: Intravenous
MedDRA: Medical Dictionary for Regulatory Activities
MeSH: Medical subject headings
NA: Not applicable
NCI: National Cancer Institute
NR: Not reported
OS: Overall survival
PDGFR: Platelet-derived growth factor receptor
PFS: Progression-free survival
PRISMA: Preferred Reporting Items for Systematic Reviews and Meta-Analyses
PS: Performance status
q3wk: Every 3 weeks
RCTs: Randomized controlled trials
STS: Soft tissue sarcoma
TTP: Time to progression
VEGFR: Vascular endothelial growth factor receptor
WHO: World Health Organization.
