# Supplementary material for: A Systematic Literature Review of Adverse Events Associated with Systemic Treatments Used in Advanced Soft Tissue Sarcoma
Source: Sarcoma. 2016 Jul 19;2016:3597609. doi: 10.1155/2016/3597609 (PMC4969544; doi:10.1155/2016/3597609)
Supplement: Supplementary file 1 — The supplemental appendix includes specific information on the literature search strategy, adverse events of interest for the comparisons, details of the identified studies included in the review, and a quality assessments of each of the articles. Also included are details of the patient characteristics of each study and the criteria used in each study to assess adverse events. [file 3597609.f1.pdf]

A systematic literature review of adverse events associated with treatments used in advanced soft tissue sarcoma

Ann Colosia[1]*, Shahnaz Khan[1], Michelle D. Hackshaw[2], Alan Oglesby[2], James A. Kaye[3] and Jeffrey M. Skolnik[4]

[1]Market Access and Outcomes Strategy, RTI Health Solutions, 200 Park Offices Drive, Research Triangle Park, NC 27709, USA; [2]US Health Outcomes, Oncology, GlaxoSmithKline, 5 Crescent Drive, Philadelphia, PA 19112, USA [3]Epidemiology, RTI Health Solutions, 1440 Main Street, Suite 310, Waltham, MA 02451, USA; [4]US Medical Affairs, Oncology, GlaxoSmithKline, 5 Crescent Drive, Philadelphia, PA 19112, USA

Supplemental Information

Table S1 PubMed search strategy for randomized controlled trials of adult patients with STS

Search number	Terms [limits: English]	Number of records
	**Disease**	
#1	((“Sarcoma”[MeSH] AND “Soft Tissue Neoplasms”[MeSH]) OR soft tissue sarcoma*[Text Word] or "Leiomyosarcoma"[Mesh] OR "leiomyosarcoma"[Text Word] OR "Sarcoma, Synovial"[MeSH] OR synovial sarcoma[Text Word]) AND (“Neoplasm Metastasis”[MeSH] OR metast*[Text Word] OR “advanced”[Text Word] OR late stage*[Text Word] OR stage 3*[Text Word] OR stage III*[Text Word] OR stage 4*[Text Word] OR stage IV*[Text Word])	6403
	**Population**	
#2	#1 AND (“Adult”[MeSH] OR "young adult"[MeSH Terms] OR "adult"[Text Word] OR ("middle aged"[MeSH Terms] OR "aged"[MeSH Terms]) OR "middle aged"[MeSH Terms] OR "aged"[MeSH Terms] OR "aged, 80 and over"[MeSH Terms OR adult*[Text Word] OR middle age*[Text Word] OR “elderly”[Text Word])	4523

	**Study type**	
#3	#2 AND (“Randomized Controlled Trial”[Publication Type] OR “Controlled Clinical Trial”[Publication Type] OR “randomized”[Title/Abstract] OR placebo*[Title/Abstract] OR “Clinical Trials as Topic”[MeSH:NoExp] OR “randomly”[Title/Abstract] OR “Randomized Controlled Trials as Topic”[MeSH] OR randomized controlled trial*[Text Word] OR randomised controlled trial*[Text Word] OR randomized clinical trial*[Text Word] OR randomised clinical trial*[Text Word] OR randomized trial*[Text Word] OR randomised trial*[Text Word] OR “random allocation”[Text Word] OR “double blind method”[Text Word] OR “single blind method”[Text Word] OR allocated random*[Text Word] OR random assignment*[Text Word] OR “randomization”[Text Word] OR “randomisation”[Text Word] OR “Double blind procedure”[Text Word] OR “Single blind procedure”[Text Word]) OR “Clinical Trial, Phase III”[Publication Type])	219
	**Exclusions**	
#4	“Animals”[MeSH] NOT “Humans”[MeSH]	3,466,588
#5	“Comment”[Publication Type] OR “Letter”[Publication Type] OR “Editorial”[Publication Type] OR clinical conference[Publication Type] OR Review[ptyp]	2,684,883
#6	#3 NOT (#4 or #5)	171
	**Drugs of interest**	
#7	#6 AND ((“pazopanib”[Text Word] OR “gemcitabine”[Text Word] OR “docetaxel”[Text Word] or "paclitaxel"[Text Word] OR “Doxorubicin”[MeSH] OR “doxorubicin”[Text Word] OR “epirubicin”[Text Word] OR “Ifosfamide”[MeSH] OR “ifosfamide”[Text Word] OR "trabectedin"[Text Word] OR "Dacarbazine"[MeSH] OR "dacarbazine"[Text Word] OR "vinorelbine"[Text Word] OR "vincristine"[Text Word] OR "temozolomide"[Text Word] OR "sunitinib"[Text Word] OR "crizotinib"[Text Word] OR "Sirolimus"[MeSH] OR "sirolimus"[Text Word] OR "bevacizumab"[Text Word] OR “sorafenib”[Text Word] OR “axitinib”[Text Word] or “imatinib”[Text Word] or “irinotecan”[Text Word] or "Topotecan"[MeSH] or “topotecan”[Text Word]))	107

MeSH, medical subject heading; STS, soft tissue sarcoma.

Table S2 List of AEs of interest

- Hematologic AEs
  - Anemia
  - Febrile neutropenia
  - Leukopenia
  - Lymphopenia
  - Neutropenia
  - Thrombocytopenia
- Liver-related AEs
  - Aspartate aminotransferase (AST) elevation
  - Alanine aminotransferase (ALT) elevation
  - Bilirubin elevation
- Gastrointestinal and/or eating-related AEs
  - Anorexia/decreased appetite
  - Constipation
  - Decreased weight or weight loss
  - Diarrhea
  - Nausea and/or vomiting
- Mouth or taste
  - Dysgeusia
  - Mucositis
- Other
  - Alopecia/hair loss
  - Asthenia
  - Cough
  - Dyspnea/shortness of breath
  - Embolism (including pulmonary and cerebrovascular)
  - Fatigue (tiredness/lack of energy)
  - Fluid retention (bloating, arm or leg swelling, edema)
  - Headache
  - Heart failure
  - Hypertension
  - Infection(s)
  - Myocardial infarction or congestive heart failure
  - Neuropathy/tingling in extremities
  - Pain (any type, including abdominal, musculoskeletal, myalgia, arthralgia)
  - Trouble sleeping/insomnia

AEs, adverse events.

Table S3 Characteristics of included studies after protocol amendment

Reference<br>Study phase	Country(ies) (number of sites)	Study period	Patients randomized, n	Treatment	Patient group for safety	n	Median number of cycles (range)
van der Graaf et al., 2012 [1]<br><br>Phase III	Australia, Europe (9 countries), Japan, South Korea, US (72 total)	10/9/2008 to 2/26/2010; and 10/24/2011 for final OS analysis	369	Pazopanib 800 mg once daily orally	Treated	239	Median treatment duration, 16.4 weeks (range, 0–79 weeks) (IQR, 6.3–30.0)
				Placebo once daily orally		123	Median treatment duration, 8.1 weeks (range, 1–52 weeks) (IQR, 4.0–13.6)
Bramwell et al., 1986, 1987, 1993 [2-4]<br><br>Crossover Phase II	Belgium, Denmark, England, Italy, the Netherlands (18 total)	Patients enrolled 10/1982-5/1984	171	Cyclophosphamide 1.5 g/m^2	Treated, analyzed, previous nonalkylating agent (except dacarbazine) chemotherapy	29	2.5 (1–13)
				Ifosfamide 5 g/m^2		28	3 (1–15)
Demetri et al., 2009 [5]<br><br>Phase II	Australia (2), Belgium (1), Canada (5), France (2), Germany (1), Italy (1), Russia (8), Spain (2), US (25)	Data through 5/31/2006 (TTP) and 4/23/2008 (survival)	270	Trabectedin 1.5 mg/m^2 24-h IV every 3 weeks	Treated	130	5 (1–37)
				Trabectedin 0.58 mg/m^2 3-h IV weekly for 3 of 4 weeks		130	2 (1–21)
García-del-Muro et al., 2011 [6]<br><br>Phase II	Spain (18)	Randomly assigned from 11/2005 to 3/2009	113	Dacarbazine 1200 mg/m^2 20-min IV every 3 weeks up to 24 weeks	Treated and assessable	52	2 (1–10)
				Gemcitabine + dacarbazine: gemcitabine 10 mg/m^2 180-min IV followed by dacarbazine 500 mg/m^2 20-min IV every 2 weeks up to 24 weeks		57	6 (2–12)

Reference<br>Study phase	Country(ies) (number of sites)	Study period	Patients randomized, n	Treatment	Patient group for safety	n	Median number of cycles (range)
Pautier et al., 2012 [7]*<br><br>Phase II	France (17)	Enrolled 2/2006 to 12/2008	46	Gemcitabine 1000 mg/m^2 fixed-dose rate of 10 mg/m^2 per min via 100-min IV on days 1, 8, and 15 every 28 days	Study 1: Uterine leiomyosarcoma, assessable	22	5
				Gemcitabine + docetaxel: Gemcitabine fixed-dose rate of 900 mg/m^2 via 90-min IV days 1 and 8; docetaxel 100 mg/m^2 via 60-min IV day 8 after gemcitabine; every 21 days with lenograstim (human recombinant G-CSF daily, 150 mcg/m^2 injection from day 9 to day 15)<br><br>If prior pelvic radiation, gemcitabine dose was 675 mg/m^2 and docetaxel dose was 75 mg/m^2<br><br>Dexamethasone was given as premedication for docetaxel: 8 mg orally, twice daily for 4 days starting the day before docetaxel administration		24	
Phase II			44	Gemcitabine, as above	Study 2: Non-uterine leiomyosarcoma assessable	22	4
				Gemcitabine + docetaxel, as above		22	

* The article by Pautier et al. [7] presents the results of two independent phase II studies: one in patients with uterine leiomyosarcoma and one in patients with non-uterine leiomyosarcoma.

G-CSF, granulocyte colony-stimulating factor; IQR, interquartile range; IV, intravenous; OS, overall survival; TTP, time to progression.

Table S4 Quality assessment of van der Graaf et al., 2012 [1]

Study Question	Grade (Yes/No/Not Clear/NA)	Notes
Was randomization carried out appropriately?	Yes	Registration was via an online randomized trial access system, and treatments were allocated with the GlaxoSmithKline online registration and medication ordering system. Patients were stratified according to number of previous lines of systemic therapy for advanced disease (0 or 1 vs. 2+) and WHO performance status (0 vs. 1). Patients were then randomly assigned with an interactive voice randomization service to receive either pazopanib 800 mg once daily or placebo (2:1), by permuted block randomization (block sizes of 6).
Was the concealment of treatment allocation adequate?	Yes	Treatment allocation remained masked until the database was locked, and the list of treatment codes was transferred to EORTC on March 1, 2011.
Were the groups similar at the outset of the study in terms of prognostic factors, for example, severity of disease?	Not clear	No statistical analyses were provided. Many of the characteristics were similar, but 73% of patients in the placebo group had high-grade tumors compared with 65% in the placebo group; 2% in the placebo group had low-grade tumors compared with 10% in the pazopanib group.
Were the care providers, participants, and outcome assessors blind to treatment allocation? If any of these people were not blinded, what might be the likely impact on the risk of bias (for each outcome)?	Yes	Patients, investigators who gave the treatment, those assessing outcomes, and those who did the analysis were masked to the allocation.
Were there any unexpected imbalances in dropouts between groups? If so, were they explained or adjusted for?	Not clear	At the data cutoff date (November 22, 2010), 94% of patients were off protocol. The median follow-up duration at the time of the primary endpoint analysis was 14.6 months in the placebo group and 14.9 months in the pazopanib group. It is not clear from the publication when AEs were assessed, but presumably because the placebo group progressed sooner than the pazopanib group (median PFS, 1.6 vs. 4.6 months), collection of AE data may have been shorter for the placebo group.
Is there any evidence to suggest that the authors measured more outcomes than they reported?	Yes	The study protocol on ClinicalTrials.gov shows other outcome measures that would rate as less clinically important than the survival outcomes presented in the publication (e.g. response rate).<br>Several of the AEs of interest for this review are listed on the ClinicalTrials.gov website and not in the article by van der Graaf et al. The laboratory values were almost completely consistent between the two sources, but the non-laboratory AE frequencies differed considerably between the online source and the publication. Therefore, we did not use the ClinicalTrials.gov results to provide non-laboratory safety data not reported in the published article.

Did the analysis include an intention-to-treat analysis? If so, was this appropriate and were appropriate methods used to account for missing data?	No ITT analysis	The safety assessments, relevant to this report, were conducted on all patients treated, which was appropriate for safety results.

AE, adverse event; EORTC, European Organisation for Research and Treatment of Cancer; ITT, intention to treat; NA, not applicable; PFS, progression-free survival; WHO, World Health Organization.
Source: National Institute for Health and Care Excellence 2009 and adapted from Centre for Reviews and Dissemination 2009.

Table S5 Quality assessment of Bramwell et al., 1986, 1987, 1993 [2-4]

Study Question	Grade (Yes/No/Not Clear/NA)	Notes
Was randomization carried out appropriately?	Not clear	Randomization methods were not described. Patients were stratified by institution and exposure (or not) to previous chemotherapy.
Was the concealment of treatment allocation adequate?	NA	Open-label study
Were the groups similar at the outset of the study in terms of prognostic factors, for example, severity of disease?	Yes	Patients were evenly distributed between the two arms with respect to age, performance status, prior chemotherapy or radiotherapy, and presence of distant metastases.
Were the care providers, participants, and outcome assessors blind to treatment allocation? If any of these people were not blinded, what might be the likely impact on the risk of bias (for each outcome)?	No	Open-label study; potential bias in reporting AEs
Were there any unexpected imbalances in dropouts between groups? If so, were they explained or adjusted for?	No	Only one patient was lost to follow-up.
Is there any evidence to suggest that the authors measured more outcomes than they reported?	Yes	The three reports give numerous outcomes by patient and disease factors for the main treatment arms. However, a selection of safety outcomes was reported.
Did the analysis include an intention-to-treat analysis? If so, was this appropriate and were appropriate methods used to account for missing data?	No ITT analysis	The study reports outcomes for evaluable patients only. This seems appropriate given that seven patients died early, two received the wrong treatment, and one was lost to follow-up. Another two patients were non-evaluable due to refusal.

AE, adverse event; ITT, intention to treat; NA, not applicable.
Source: National Institute for Health and Care Excellence 2009 and adapted from Centre for Reviews and Dissemination 2009.

Table S6 Quality assessment of Demetri et al., 2009 [5]

Study Question	Grade (Yes/No/Not Clear/NA)	Notes
Was randomization carried out appropriately?	Yes	Permuted-block randomization with stratification by baseline ECOG PS score of 0 or 1
Was the concealment of treatment allocation adequate?	NA	Open-label study
Were the groups similar at the outset of the study in terms of prognostic factors, for example, severity of disease?	Not clear	No statistical analyses were provided. Many of the characteristics were similar, but the weekly 3-hour group had 96.3% with metastatic disease vs. 89.7% in the every 3 weeks 24-hour group
Were the care providers, participants, and outcome assessors blind to treatment allocation? If any of these people were not blinded, what might be the likely impact on the risk of bias (for each outcome)?	No	Open-label study; potential bias in reporting AEs
Were there any unexpected imbalances in dropouts between groups? If so, were they explained or adjusted for?	No	Apart from disease progression, 34 patients permanently discontinued treatment in the every 3 weeks 24-hour arm compared with 37 patients in the weekly 3-hour arm
Is there any evidence to suggest that the authors measured more outcomes than they reported?	Yes	The usual outcomes were reported (TTP, PFS, OS, subanalyses of these, and safety). However, a selection of safety outcomes was reported.
Did the analysis include an intention-to-treat analysis? If so, was this appropriate and were appropriate methods used to account for missing data?	No ITT analysis	Only treated patients were included in the analysis, and this seemed appropriate.

AE, adverse event; ECOG, Eastern Cooperative Oncology Group; ITT, intention to treat; NA, not applicable; OS, overall survival; PFS, progression-free survival; PS, performance status; TTP, time to progression.
Source: National Institute for Health and Care Excellence 2009 and adapted from Centre for Reviews and Dissemination 2009.

Table S7 Quality assessment of García-del-Muro et al., 2011 [6]

Study Question	Grade (Yes/No/Not Clear/NA)	Notes
Was randomization carried out appropriately?	Not clear	Randomization method not described. Assignment was stratified by ECOG PS (0–1 vs. 2) and interval from the initial diagnosis to first relapse (≤12 months vs. >12 months)
Was the concealment of treatment allocation adequate?	No	Open-label study
Were the groups similar at the outset of the study in terms of prognostic factors, for example, severity of disease?	Not clear	No statistical analyses were provided. Several characteristics differed by more than a few percentage points for the gemcitabine + dacarbazine group vs. the dacarbazine group:<br>▪ One involved site: 49% vs. 39%<br>▪ ECOG PS 0: 39% vs. 33%<br>▪ ECOG PS 1: 53% vs. 60%<br>▪ Primary tumor in extremity and trunk wall: 40% vs. 52%
Were the care providers, participants, and outcome assessors blind to treatment allocation? If any of these people were not blinded, what might be the likely impact on the risk of bias (for each outcome)?	No	Open-label study; potential bias in reporting AEs
Were there any unexpected imbalances in dropouts between groups? If so, were they explained or adjusted for?	No	Most patients discontinued due to disease progression, although somewhat fewer in the combination therapy arm. The number of dropouts for “toxicity” and “other” were similar in the two treatment groups.
Is there any evidence to suggest that the authors measured more outcomes than they reported?	Yes	The usual outcomes were reported (TTP, PFS, OS, subanalyses of these, and safety). However, only safety outcomes considered by the authors to be clinically relevant were reported.
Did the analysis include an intention-to-treat analysis? If so, was this appropriate and were appropriate methods used to account for missing data?	No ITT analysis	Only patients who were treated and analyzed were considered in the efficacy and safety results. This seems appropriate, because the excluded patients were not eligible (n = 3) or withdrew consent (n = 1).

AE, adverse event; ECOG, Eastern Cooperative Oncology Group; ITT, intention to treat; NA, not applicable; OS, overall survival; PFS, progression-free survival; PS, performance status; TTP, time to progression.
Source: National Institute for Health and Care Excellence 2009 and adapted from Centre for Reviews and Dissemination 2009.

Table S8 Quality assessment of Pautier et al., 2012, uterine leiomyosarcoma [7]

**Study Question**	**Grade (Yes/No/Not Clear/NA)**	**Notes***
Was randomization carried out appropriately?	Not clear	No details were reported about the randomization method, in the article or on ClincialTrials.gov.
Was the concealment of treatment allocation adequate?	No	Open-label study
Were the groups similar at the outset of the study in terms of prognostic factors, for example, severity of disease?	No	In the uterine groups, higher proportions of patients had received first-line anthracycline-based chemotherapy (95%) and radiation (73%) in the gemcitabine group compared with the gemcitabine plus docetaxel group (75% and 50%, respectively).
Were the care providers, participants, and outcome assessors blind to treatment allocation? If any of these people were not blinded, what might be the likely impact on the risk of bias (for each outcome)?	No	Open-label study; potential bias in reporting AEs
Were there any unexpected imbalances in dropouts between groups? If so, were they explained or adjusted for?	No	Four patients in the uterine groups were excluded due to major protocol violations. One death occurred due to toxicity in the combination therapy arm, although it was not clear whether this patient had uterine or non-uterine leiomyosarcoma.
Is there any evidence to suggest that the authors measured more outcomes than they reported?	Yes	AEs were reported only by percentage of cycles, not percentage of patients.
Did the analysis include an intention-to-treat analysis? If so, was this appropriate and were appropriate methods used to account for missing data?	Not clear	Survival was calculated by ITT and by evaluability. The size of the safety population is not clear, because the AEs are presented as percentages by cycle with no sample sizes given.

* Answers apply to both phase II studies included in this article unless otherwise noted.
AE, adverse event; ITT, intention to treat; NA, not applicable.
Source: National Institute for Health and Care Excellence 2009 and adapted from Centre for Reviews and Dissemination 2009.

Table S9 Quality assessment of Pautier et al., 2012, non-uterine leiomyosarcoma [7]

Study Question	Grade (Yes/No/Not Clear/NA)	Notes*
Was randomization carried out appropriately?	Not clear	No details were reported about the randomization method, in the article or on ClincialTrials.gov.
Was the concealment of treatment allocation adequate?	No	Open-label study
Were the groups similar at the outset of the study in terms of prognostic factors, for example, severity of disease?	No	In the non-uterine groups, higher proportions of patients had receiving first-line anthracycline-based chemotherapy (82%) and radiation therapy (50%) in the gemcitabine plus docetaxel group compared with the gemcitabine group (77% and 43%, respectively).
Were the care providers, participants, and outcome assessors blind to treatment allocation? If any of these people were not blinded, what might be the likely impact on the risk of bias (for each outcome)?	No	Open-label study; potential bias in reporting AEs
Were there any unexpected imbalances in dropouts between groups? If so, were they explained or adjusted for?	No	Three patients in the non-uterine groups were excluded due to major protocol violations. One death occurred due to toxicity in the combination therapy arm, although it was not clear whether this patient had uterine or non-uterine leiomyosarcoma.
Is there any evidence to suggest that the authors measured more outcomes than they reported?	Yes	AEs were reported only by percentage of cycles, not percentage of patients.
Did the analysis include an intention-to-treat analysis? If so, was this appropriate and were appropriate methods used to account for missing data?	Not clear	Survival was calculated by ITT and by evaluability. The size of the safety population is not clear, because the AEs are presented as percentages by cycle with no sample sizes given.

* Answers apply to both phase II studies included in this article unless otherwise noted.
AE, adverse event; ITT, intention to treat; NA, not applicable.
Source: National Institute for Health and Care Excellence 2009 and adapted from Centre for Reviews and Dissemination 2009.

Table S10 Patient characteristics

<table>
<tr><th rowspan="2">Reference</th><th rowspan="2">Previous systemic anticancer therapy criteria/experience</th><th rowspan="2">Treatment</th><th rowspan="2">Median age (range); female; performance status</th><th rowspan="2">Metastatic disease; advanced</th><th colspan="3">Previous therapy</th></tr>
<tr><th>Rad</th><th>Adjuv</th><th>Chemo</th></tr>
<tr><td rowspan="4">van der Graaf et al., 2012 [1, 8]</td><td rowspan="4">▪ Previous adjuvant or neoadjuvant therapy: 94 (25%)
▪ Previous systemic therapy for advanced disease: 342 (93%)
▪ ≥2 lines of treatment: 207 (56%)
▪ ≥3 lines of treatment: 78 (21%)</td><td rowspan="2">Pazopanib</td><td colspan="5">STS Histology
Total: 246 (100%); Leiomyosarcoma: 109 (44.3%); Synovial sarcoma: 25 (10.2%); Other STS histologies: 112 (45.5%)</td></tr>
<tr><td>56.7 (20.1–83.7) y
Female: 60%
WHO PS 0: 46%
WHO PS 1: 54%</td><td>246 (100%);
0 (0%)</td><td>NR</td><td>NR</td><td>246 (100%)</td></tr>
<tr><td rowspan="2">Placebo</td><td colspan="5">STS Histology
Total: 123 (100%); Leiomyosarcoma: 49 (39.8%); Synovial sarcoma: 13 (10.6%); Other STS histologies: 61 (49.6%)</td></tr>
<tr><td>51.9 (18.8–78.6) y
Female: 56%
WHO PS 0: 46%
WHO PS 1: 54%</td><td>123 (100%);
0 (0%)</td><td>NR</td><td>NR</td><td>123 (100%)</td></tr>
<tr><td rowspan="3">Bramwell et al., 1986, 1987, 1993 [2-4]</td><td rowspan="3">Excluded if previous classic alkylating agent except dacarbazine
Of 135 total patients, 57 patients, 1 drug: 68%;
≥3 drugs: 12.5%</td><td rowspan="2">Cyclophosphamide</td><td colspan="5">STS Histology
Total: 67 (100%); Leiomyosarcoma: 15 (22.4%); Synovial sarcoma: 8 (11.9%); Fibrosarcoma: 3 (4.5%); Malignant fibrous histiocytoma: 3 (4.5%); Undifferentiated: 5 (7.5%); Neurofibrosarcoma: 3 (4.5%); Liposarcoma: 2 (3.0%); Angiosarcoma: 1 (1.5%); Rhabdomyosarcoma: 0 (0%); Unclassified: 7 (10.5%); Mixed mesodermal sarcoma: 5 (7.5%); Endometrial stromal sarcoma: 3 (4.5%); Miscellaneous: 4 (6.0%); Untested: 8 (11.9%)</td></tr>
<tr><td>47 (NR) y
Female: 45%
WHO 0–1: 76%
(PS 0–2 included)</td><td>61 (91%)</td><td>21 (31%)</td><td>NR</td><td>29 (43%)</td></tr>
<tr><td>Ifosfamide</td><td colspan="5">STS Histology
Total: 68 (100%); Leiomyosarcoma: 16 (23.5%); Synovial sarcoma: 9 (13.2%); Fibrosarcoma: 4 (5.9%); Malignant fibrous histiocytoma: 4 (5.9%); Undifferentiated: 2 (2.9%); Neurofibrosarcoma: 2 (2.9%); Liposarcoma: 2 (2.9%); Angiosarcoma: 2 (2.9%); Rhabdomyosarcoma: 2 (2.9%); Unclassified: 2</td></tr>
</table>

Reference	Previous systemic anticancer therapy criteria/experience	Treatment	Median age (range); female; performance status	Metastatic disease; advanced	Previous therapy		
					Rad	Adjuv	Chemo
			(2.9%); Mixed mesodermal sarcoma: 8 (11.8%); Endometrial stromal sarcoma: 2 (2.9%); Miscellaneous: 1 (1.5%); Untested: 12 (17.6%)				
			49 (NR) y<br>Female: 59%<br>WHO 0–1: 79%	62 (91%)	21 (31%)	NR	28 (41%)
Demetri et al., 2009 [5]	Inclusion criteria:<br>▪ At least previous anthracycline and ifosfamide (combined or sequential)<br>▪ Median prior chemotherapeutic drugs: 3<br>▪ Median previous regimens: 2<br>▪ Median previous lines of therapy for advanced disease: 1 (range, 0–6)	Trabectedin q3wk 24-h IV	*STS Histology*<br>Total: 136 (100%); Leiomyosarcoma: 72 (52.9%); Liposarcoma: 30 (22.1%); Other: 15 (11.0%); Missing: 19 (14.0%)				
			53 (20–80) y<br>Female: 67.6%<br>ECOG 0: 51.5%<br>ECOG 1: 48.5%	122 (89.7%); 13 (9.6%)	71 (52.2%)	NR	136 (100%)
		Trabectedin weekly 3-h IV	*STS Histology*<br>Total: 134 (100%); Leiomyosarcoma: 66 (49.3%); Liposarcoma: 45 (33.6%); Other: 8 (6.0%); Missing: 15 (11.2%)				
			54 (23–77) y<br>Female: 58.2%<br>ECOG PS 0: 50%<br>ECOG PS 1: 50%	129 (96.3%); 4 (3.0%)	68 (50.7%)	NR	134 (100%)
García-del-Muro et al., 2011 [6]	Inclusion criteria: unresectable or metastatic progressive disease after prior treatment with anthracyclines and ifosfamide, or contraindications for their use<br>▪ Prior anthracyclines: 107<br>– With ifosfamide: 78<br>▪ Prior ifosfamide only: 2	Dacarbazine	*STS Histology*<br>Total: 52 (100%); Leiomyosarcoma: 16 (31%); Liposarcoma: 9 (17%); Undifferentiated pleomorphic: 8 (15%); Synovial sarcoma: 5 (10%); Miscellaneous sarcoma: 14 (27%)				
			51 (25–73) y<br>Female: 46%<br>ECOG PS 0: 33%<br>ECOG PS 1: 60%<br>ECOG PS 2: 8%	48 (92%); 4 (8%)	NR	NR	52 (100%)
		Gemcitabine + dacarbazine	*STS Histology*<br>Total: 57 (100%); Leiomyosarcoma: 16 (28%); Liposarcoma: 10 (18%); Undifferentiated pleomorphic: 11 (19%); Synovial sarcoma: 6 (11%); Miscellaneous sarcoma: 14 (25%)				

Reference	Previous systemic anticancer therapy criteria/experience	Treatment	Median age (range); female; performance status	Metastatic disease; advanced	Previous therapy		
					Rad	Adjuv	Chemo
			49 (18–78) y<br>Female: 47%<br>ECOG PS 0: 39%<br>ECOG PS 1: 53%<br>ECOG PS 2: 9%	50 (88%);<br>7 (12%)	NR	NR	57 (100%)
Pautier et al., 2012 [7]	Inclusion criteria: only 1 previous doxorubicin-containing regimen<br>▪ First-line anthracycline-based chemotherapy: 21<br>▪ Prior adjuvant <1 year before relapse: 1<br>▪ Prior adjuvant >1 year before relapse (ineligible for efficacy analysis): 1	Gemcitabine	*STS Histology*<br>Uterine leiomyosarcoma: 22 (100%)				
			54 (41–80) y<br>Female: 100%<br>ECOG PS, median 0 (range, 0–2)	22 (100%);<br>NR	16 (73%)	2 (9%)	22 (100%)
	▪ First-line anthracycline-based chemotherapy: 18<br>▪ Prior adjuvant <1 year before relapse: 6<br>▪ Prior adjuvant >1 year before relapse (ineligible for efficacy analysis): 3	Gemcitabine + docetaxel	*STS Histology*<br>Uterine leiomyosarcoma: 24 (100%)				
			58 (43–76) y<br>Female: 100%<br>ECOG PS, median 0 (range, 0–2)	23 (96%);<br>NR	12 (50%)	9 (38%)	24 (100%)

Reference	Previous systemic anticancer therapy criteria/experience	Treatment	Median age (range); female; performance status	Metastatic disease; advanced	Previous therapy		
					Rad	Adjuv	Chemo
			STS Histology<br>Non-uterine leiomyosarcoma: 22 (100%)				
	▪ First-line anthracycline-based chemotherapy: 17<br>▪ Prior adjuvant <1 year before relapse: 5<br>▪ Prior adjuvant >1 year before relapse: 0	Gemcitabine	64 (35–74) y<br>Female: 45%<br>ECOG PS, median 1 (range, 0–2)	20 (91%)	9 (43%)	5 (23%)	22 (100%)
			STS Histology<br>Non-uterine leiomyosarcoma: 22 (100%)				
	▪ First-line anthracycline-based chemotherapy: 18<br>▪ Prior adjuvant <1 year before relapse: 4<br>▪ Prior adjuvant >1 year before relapse (ineligible for efficacy analysis): 2	Gemcitabine + docetaxel	62 (29–78) y<br>Female: 59%<br>ECOG PS, median 1 (range, 0–2)	21 (95%)	11 (50%)	6 (27%)	22 (100%)

Adjuv, adjuvant therapy; Chemo, chemotherapy; ECOG, Eastern Cooperative Oncology Group; IV, intravenous; NR, not reported; PS, performance status; Rad, radiation therapy; STS, soft tissue sarcoma; WHO, World Health Organization; y, years.

Table S11 Type of AEs reported in included studies

**Study**	van der Graaf et al., 2012 [1, 8]	Bramwell et al., 1986, 1987, 1993 [2-4]	Demetri et al., 2009 [5]	García-del-Muro et al., 2011[6]	Pautier et al., 2012 [7]
**Interventions**	Pazopanib<br>Placebo	Cyclophosphamide<br>Ifosfamide	Trabectedin q3wk 24-h IV<br>Trabectedin weekly 3-h IV	Dacarbazine<br>Gemcitabine + dacarbazine	Gemcitabine<br>Gemcitabine + docetaxel
**Definition**	NCI CTCAE v 3.0	WHO grade toxicity	MedDRA v 8.0; grade by NCI CTC v 2.0	NCI CTC v 3.0	NCI CTC (v, NR)
**Time Frame**	Safety assessment was from baseline (day 1) until the study drug was discontinued or the treatment period ended; mean assessment duration was 20 weeks for laboratory data	"After the first course and throughout treatment"<br>Median cycles (range) for all (with and without previous treatment) patients:<br>▪ Cyclophosphamide: 2.5 (1–13) (3-week cycles)<br>▪ Ifosfamide: 3.0 (1–15) (3-week cycles)	"All patients were followed until recovery from toxicity"<br>No other time frame reported<br>Median cycles (range):<br>▪ Q3wk 24-h infusion schedule: 5 (1–37)<br>▪ Weekly 3-h infusion schedule: 2 (1–21)	No time frame reported<br>Median cycles (range):<br>▪ Dacarbazine: 2 (1–10) (3-week cycles)<br>▪ Gemcitabine + dacarbazine: 6 (2–12) (2-week cycles)	No time frame reported<br>Median cycles of 3 to 4 weeks:<br>▪ Uterine leiomyosarcoma: 5<br>▪ Non-uterine leiomyosarcoma: 4
**Reported AEs**	Common treatment-emergent AEs and shift from baseline in laboratory assessments	Only leukopenia was reported for the subgroup previously treated with chemotherapy	Non-laboratory values: Most common trabectedin-related worst grade AE (≥10% of patients) reaching grade 3 or 4 in either treatment arm<br>Laboratory values: Worst on-treatment laboratory abnormalities (not clear if drug related)	Worst grade of clinically relevant toxicities	Percentage of cycles for which patients experienced toxicity
**Outcome**	n (%) of patients	n (%) of patients	n (%) of patients<br>n (%) of cycles during which AE was experienced	% of patients	% of cycles during which AE was experienced

AE, adverse event; CTC, Common Terminology Criteria; CTCAE, Common Terminology Criteria for Adverse Events; IV, intravenous; MedDRA, *Medical Dictionary for Regulatory Activities*; NCI, National Cancer Institute; NR, not reported; q3wk, every 3 weeks; WHO, World Health Organization.

References

[1] W. T. van der Graaf, J. Y. Blay, S. P. Chawla, D. W. Kim, B. Bui-Nguyen, P. G. Casali, P. Schoffski, M. Aglietta, A. P. Staddon, Y. Beppu, A. Le Cesne, H. Gelderblom, I. R. Judson, N. Araki, M. Ouali, S. Marreaud, R. Hodge, M. R. Dewji, C. Coens, G. D. Demetri, C. D. Fletcher, A. P. Dei Tos, P. Hohenberger, E. S. Tissue, G. Bone Sarcoma, and P. s. group, "Pazopanib for metastatic soft-tissue sarcoma (PALETTE): a randomised, double-blind, placebo-controlled phase 3 trial," *Lancet.* vol. 379, no. 9829, pp. 1879-1886, 2012.

[2] V. H. Bramwell, H. T. Mouridsen, A. Santoro, G. Blackledge, R. Somers, D. Thomas, R. Sylvester, and A. Van Oosterom, "Cyclophosphamide versus ifosfamide: preliminary report of a randomized phase II trial in adult soft tissue sarcomas," *Cancer Chemother Pharmacol.* vol. 18 Suppl 2, no. pp. S13-16, 1986.

[3] V. H. Bramwell, H. T. Mouridsen, A. Santoro, G. Blackledge, R. Somers, J. Verwey, P. Dombernowsky, M. Onsrud, D. Thomas, R. Sylvester, and A. van Oosterom, "Cyclophosphamide versus ifosfamide: final report of a randomized phase II trial in adult soft tissue sarcomas," *Eur J Cancer Clin Oncol.* vol. 23, no. 3, pp. 311-321, 1987.

[4] V. H. Bramwell, H. T. Mouridsen, A. Santoro, G. Blackledge, R. Somers, J. Verweij, P. Dombernowsky, M. Onsrud, D. Thomas, R. Sylvester, and et al., "Cyclophosphamide versus ifosfamide: a randomized phase II trial in adult soft-tissue sarcomas. The European Organization for Research and Treatment of Cancer [EORTC], Soft Tissue and Bone Sarcoma Group," *Cancer Chemother Pharmacol.* vol. 31 Suppl 2, no. pp. S180-184, 1993.

[5] G. D. Demetri, S. P. Chawla, M. von Mehren, P. Ritch, L. H. Baker, J. Y. Blay, K. R. Hande, M. L. Keohan, B. L. Samuels, S. Schuetze, C. Lebedinsky, Y. A. Elsayed, M. A. Izquierdo, J. Gomez, Y. C. Park, and A. Le Cesne, "Efficacy and safety of trabectedin in patients with advanced or metastatic liposarcoma or leiomyosarcoma after failure of prior anthracyclines and ifosfamide: results of a randomized phase II study of two different schedules," *J Clin Oncol.* vol. 27, no. 25, pp. 4188-4196, 2009.

[6] X. Garcia-Del-Muro, A. Lopez-Pousa, J. Maurel, J. Martin, J. Martinez-Trufero, A. Casado, A. Gomez-Espana, J. Fra, J. Cruz, A. Poveda, A. Meana, C. Pericay, R. Cubedo, J. Rubio, A. De Juan, N. Lainez, J. A. Carrasco, R. de Andres, J. M. Buesa, and S. Spanish Group for Research on, "Randomized phase II study comparing gemcitabine plus dacarbazine versus dacarbazine alone in patients with previously treated soft tissue sarcoma: a Spanish Group for Research on Sarcomas study," *J Clin Oncol.* vol. 29, no. 18, pp. 2528-2533, 2011.

[7] P. Pautier, A. Floquet, N. Penel, S. Piperno-Neumann, N. Isambert, A. Rey, E. Bompas, A. Cioffi, C. Delcambre, D. Cupissol, F. Collin, J. Y. Blay, M. Jimenez, and F. Duffaud, "Randomized multicenter and stratified phase II study of gemcitabine alone versus gemcitabine and docetaxel in patients with metastatic or relapsed leiomyosarcomas: a Federation Nationale des Centres de Lutte Contre le Cancer (FNCLCC) French Sarcoma Group Study (TAXOGEM study)," *Oncologist.* vol. 17, no. 9, pp. 1213-1220, 2012.

[8] ClinicalTrials.gov. Pazopanib Versus Placebo in Patients With Soft Tissue Sarcoma Whose Disease Has Progressed During or Following Prior Therapy (PALETTE). http://clinicaltrials.gov/ct2/show/NCT00753688?term=00753688&rank=1 (accessed October 6 2014).
